# Lacrimal ductal cyst of the medial orbit: a case report

**DOI:** 10.1186/s12886-020-01636-1

**Published:** 2020-09-24

**Authors:** Yu Zhang, Changhong Zeng, Ningshao Chen, Chunling Liu

**Affiliations:** Department of Ophthalmology, West China Hospital, Sichuan University, Chengdu, 610041 Sichuan People’s Republic of China

**Keywords:** Lacrimal ductal cyst, Dacryops, Intraorbital mass, Intraocular pressure

## Abstract

**Background:**

The lacrimal ductal cyst (dacryops) is an uncommon clinical entity. It occurs anywhere that lacrimal gland tissue is present but most often appears as an expanding mass in the region of the lacrimal gland. The presence involving the medial part of the orbit is rare, ectopic location can be misleading in the differential diagnosis of orbital masses. The authors report a 53-year-old man who presented with dacryops occurred in an unusual location with significant clinical presentations.

**Case presentation:**

A 53-year-old man had a painless mass located in the right superomedial orbit accompanied with foreign body sensation and lachrymation for two months, which had rapidly grown within 10 days. Decrease of visual acuity, high intraocular pressure (IOP) and limitation of extraocular movements in the right eye were present. The result of visual evoked potential (VEP) test suggested the impaired function of the optic nerve. Magnetic resonance imaging (MRI) studies revealed the presence of an isolated cystic lesion. The mass was completely removed via a transcutaneous approach, histopathologic findings were consistent with the lacrimal ductal cyst. The ocular motility and high IOP returned to normal. There had been no post-operative complications or signs of recurrence over five months follow-up.

**Conclusion:**

Lacrimal ductal cysts can present in the medial orbit, clinicians should include this entity in the differential diagnosis of orbital masses and be aware of its variable presentations such as high IOP in this case. We comment on the fact that many reported cases of ectopic dacryops may be an extension of normal lacrimal gland tissue.

## Background

The lacrimal ductal cyst (dacryops) is a benign epithelial tumor of the lacrimal tissue, which usually locates in the outer part of the upper eyelid presenting to be an asymptomatic, smooth, mobile and fluctuant swelling [1]. Ectopic dacryops is lacrimal ductal cyst located at any site other than the normal. The presence involving the medial part of the orbit is very rare [1–4]. These cysts may cause mechanical blepharoptosis, proptosis, mechanical restriction of the ocular motility and diplopia [5, 6]. Secondary high intraocular pressure due to dacryops has not been reported previously. Herein, the authors report a case of ectopic dacryops in the medial orbit.

## Case presentation

**Fig. 1:**
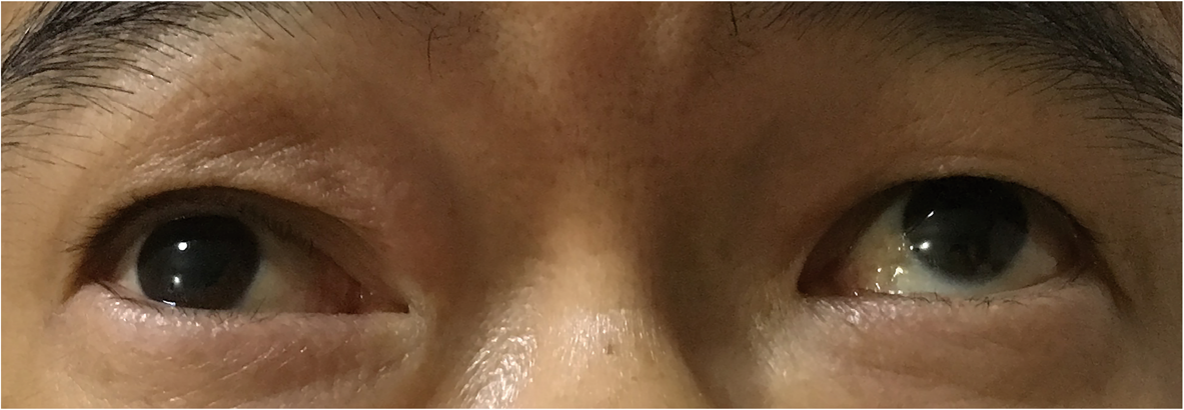
External appearance. A 53-year-old man with a painless mass in the right superior medial orbit. The right eye had difficulty in superior motility

**Fig. 2:**
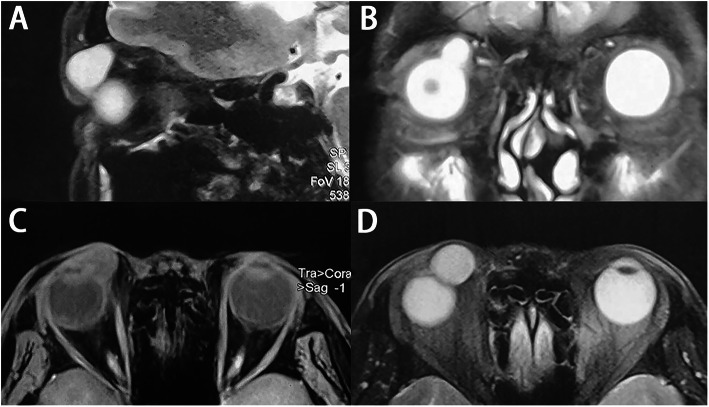
Magnetic resonance imaging shows a fluid-density cyst in the right superior medial orbit. **a**, Sagittal plane, **b**, Coronal plane, **c**, Axial plane T1weighted, **d**, Axial plane T2weighted

**Fig. 3:**
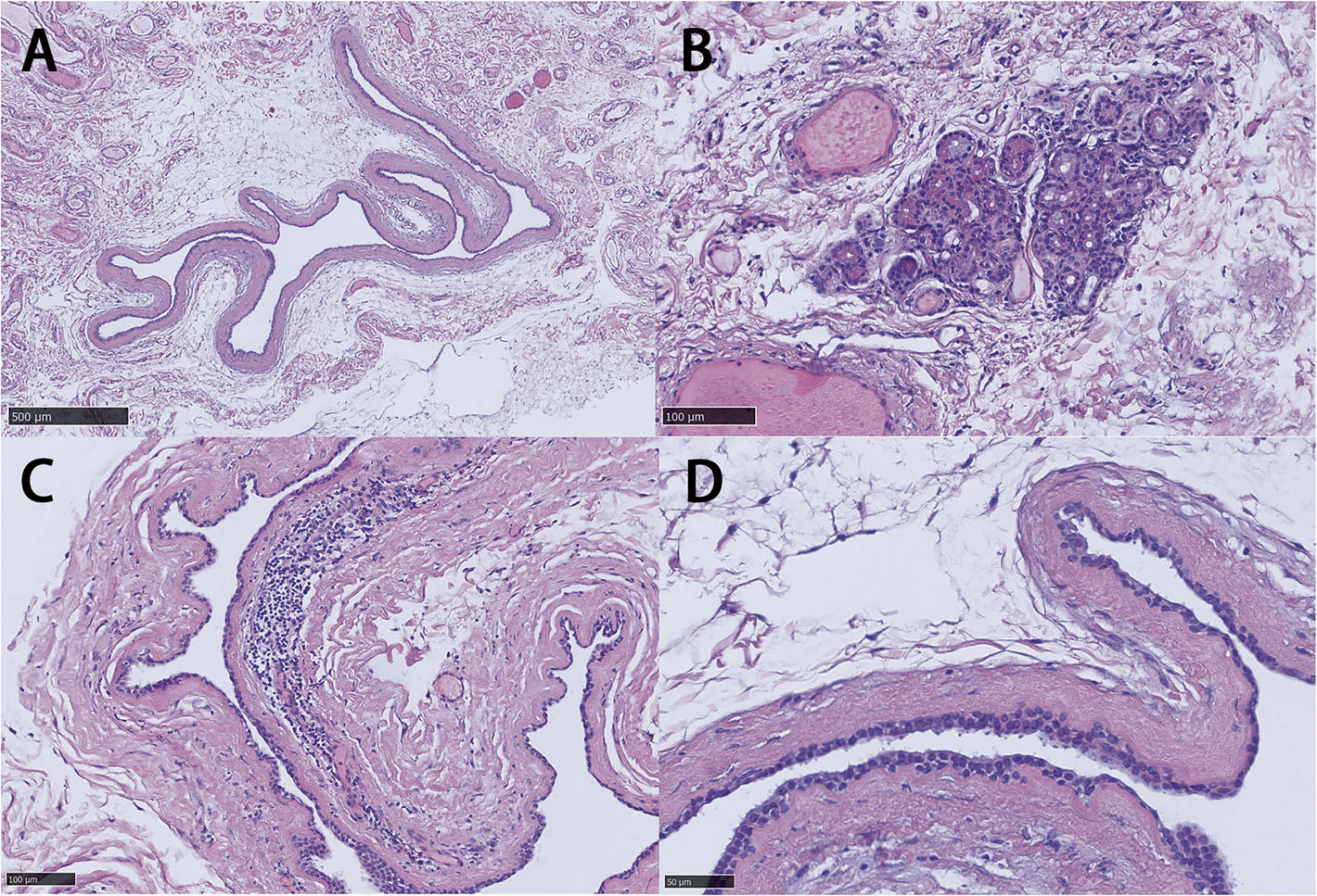
Histopathology of the dacryops. **a**, A large cystic area lined with two layers of cuboidal epithelium similar to that of lacrimal ductal structures. Note absence of Goblet cells (hematoxylin and eosin staining, original magnification, × 50). **b**, Glandular tissue (hematoxylin and eosin staining, original magnification, × 200). **c**, Adjacent to the cyst wall contains a mild to moderate chronic inflammatory cell infiltration (hematoxylin and eosin staining, original magnification, × 200). **d**, The apical changes of apocrine secretion. (hematoxylin and eosin staining, original magnification, × 400)

A 53-year-old man complained of a painless swelling of the right upper lid for 2 months accompanied with foreign body sensation and lachrymation. The mass had rapidly developed within 10 days, which could be enlarged and purplish blue after rubbing the eye. The patient denied the history of trauma, infection or surgery. A cystic mass could be felt at the superior-medial of the orbit, which was smooth, mobile, fluctuant and without tenderness. There was some underactive motility when the patient looked upward (Fig. 1). No mass was evident on everting the upper lid. The anterior segment and fundus examination were normal. The corrected visual acuity in the right eye was 12/20 and was 20/20 in the left eye, the intraocular pressure (IOP) is 36 mmHg and 18 mmHg respectively. The anterior segment and fundus examination were normal. Visual evoked potential (VEP) test showed significant delay of the P100 component peak time in the right eye for 19.8 ms. Axial magnetic resonance imaging (MRI) studies revealed the presence of an isolated 16.2 × 13.7 mm cystic lesion occupying the superior right orbit, adjacent to the trochlea and superior oblique muscle. The content of the cyst showed medium intensity on T1 and hyperintensity T2 weighted imaging (Fig. 2). Under general anesthesia, we performed a surgical excision through a transcutaneous approach. Intraoperatively, the cyst was found extending posteriorly behind the orbital septum, with mild adhesion to surrounding tissues. It ruptured during removal separation towards the conjunctiva and spilled out clear, colourless, thin watery fluid, similar to tears. The cyst wall was completely excised and a reconstructive conjunctivoplasty was performed. The specimen consisted of a collapsed cyst measuring 18 mm × 10 mm × 5 mm, the cyst wall was about 0.1–0.2 mm. On light microscopy, the surface of the capsule was lined with double cubic epithelium. Noticeable apocrine differentiation was evident as the epithelium seems to present villous projections into the lumen at its apex. Adjacent to the cyst wall contained a mild to moderate chronic inflammatory cell infiltration. Lacrimal gland tissue was found. (Fig. 3) The final diagnosis was consistent with a cyst of ductal origin. The first day after the operation, the corrected visual acuity was 20/20 in both eyes, the IOP was 18.2 mmHg in the right eye and 18.4 mmHg in the left eye, the ocular motility returned to normal. There had been no post-operative complications or signs of recurrence over a five-month follow-up.

## Discussion and conclusion

Lacrimal ductal cyst is not common, from studies in Wills Eye Hospital by Shields et al.,it accounts for up to 2% of all orbital mass and 6% of lacrimal gland lesions [7, 8]. The term dacryops for cysts of the palpebral lobe was introduced by Schmidt in 1803, which has been extended to describe lacrimal gland cysts of other kinds. Typically, they present at the superotemporal eyelid fornix where the most lacrimal gland tissue is present. Bullock proposed a classification system based on the location of the lacrimal gland tissue: palpebral lobe cysts (simple dacryops); orbital lobe cysts; cysts of the accessory lacrimal glands of Krause and Wolfring; and cysts of ectopic (choristomatous) lacrimal glands [1].

Among all forms of dacryops from the literature, simple dacryops is the most common which often protrude into the lateral upper fornix [1, 9, 10]. Orbital lobe cysts tend to present in infancy and early childhood, deep in the orbit producing proptosis and inferonasal displacement of the globe [1, 3]. Krause glands located within the fornix, while Wolfring glands are adherent or adjacent to the proximal tarsal border of the eyelids. Cysts of accessory lacrimal gland are generally to be found in the conjunctiva of the fornix [11, 12]. Ectopic lacrimal gland tissue has been reported to occur in in the eyelid, outer canthus, conjunctiva, sclera, caruncle, cornea, iris, choroid,and the orbit [1, 2, 11]. Alyahya et al. reported the largest series of ectopic lacrimal gland describing 61 such cases, all the cases were located temporally [13] . The presence involving the medial part of the orbit is rare. It is difficult to firstly consider the lacimal gland origin when a cyst occurs in the medial orbit. Clues to the diagnosis are both anatomic and histopathologic [11, 14]. However, in this patient it is difficult to distinguish ectopic dacryops with accessory lacrimal gland cysts for the histopathologic appearance is identical regardless of origin and location [15]. It was seldom diagnosed accurately for many reports in the literature were both confusing and inaccurate. There was a similar case of ectopic dacryops above the orbit was later thought to be a Wolfring’s accessory lacrimal gland cyst [11, 16]. Many other authors share this same opinion that many reported cases of ectopic dacryops might be an extension of normal lacrimal gland tissue [17].. By definition, we believe the lesion in this patient is ectopic dacryops because of the unusual anatomic location in the deep orbit and the histological assessment. Nevertheless, whether the possible source is the accessory lacrimal gland or ectopic lacrimal tissue (choristomas) is difficult to say.

The pathogenesis for the development of dacryops is still unclear. It has been reported associated with trachoma, conjunctival inflammation, ocular cicatricial pemphigoid, high levels of IgA, prolapse of the lacrimal gland, ocular trauma and chemical injury [11, 15, 18]. Initial reports proposed mechanical blockage theory and impaired ductal myoepithelial contractility, others proposed that a combination of periductal inflammation and trauma and a dysfunction of the rich neural plexus and hypersecretion may cause cyst formation [12, 19, 20]. The findings of apocrine changes, lymphoid tissue and lachrymation in this case may help to explain the pathogenesis of the disease.

Dacryops is usually asymptomatic and insidious, which can grow rapidly in the context of infection [21].. The chronic progression is capable of undergoing malignant transformation [22].. There are no definite guidelines for treatment. Most reports recommend observation if the tumor is silent and the cysts are not progressively enlarging. The indications for surgical treatment include diplopia, blepharoptosis, pain, chronic irritation or infection [21]. The clinical manifestations in this case included tears, limitation of extraocular movements, decrease of visual acuity, high intraocular pressure, and rapid growth of dacryops. With the mass grew rapidly, short-term high IOP resulted to early impairment of the innermost retinal layers, notwithstanding a normal optic disc and visual field analysis [23]. The examination first presented the latency of P100 waves. The compression of optic nerve was revealed by the VEP abnormalities in this case, which might be overlooked in the past. The rapid progress and significantly increased IOP prompted the authors to perform the surgery, so as to confirm the diagnosis and avoid further optic nerve injury. Considering the mass was located in the anterior superior medial orbit close to the trochlea, the author took an orbitotomy through a transcutaneous approach. The ocular motility, visual acuity and high intraocular pressure returned to normal after the operation. Complete excision of dacryops is generally recommended to reduce the risk of recurrence [11, 12, 15].

In conclusion, we describe an uncommon lacrimal ductal cyst in an atypical location, which should be considered in the differential diagnosis of medial orbital masses. Although the dacryops presents as benign tumor, the mass effects deserve attention for treatment.

## Data Availability

Not applicable.

## Abbreviations

IOP: Intraocular pressure
VEP: Visual evoked potential
MRI: Magnetic resonance imaging
